# Aortic arch replacement with frozen elephant trunk technique – a single-center study

**DOI:** 10.1186/s13019-019-0969-9

**Published:** 2019-08-01

**Authors:** Jamila Kremer, Fabian Preisner, Bashar Dib, Ursula Tochtermann, Arjang Ruhparwar, Matthias Karck, Mina Farag

**Affiliations:** 1 Department of Cardiac Surgery, Heidelberg University Hospital, Im Neuenheimer Feld 110, 69120 Heidelberg, Germany; 2 Department of Neuroradiology, Heidelberg University Hospital, Im Neuenheimer Feld, 400 Heidelberg, Germany

**Keywords:** Aortic disease, Frozen elephant trunk, Follow-up downstream aorta

## Abstract

**Background:**

The frozen elephant trunk (FET) technique was developed to facilitate the two-stage surgery of extensive pathologies of the thoracic aorta and is now routinely applied in acute and chronic aortic syndromes.

**Methods:**

From 11/2006 to 07/2017, 68 patients underwent aortic arch repair using the FET technique. Patients received either the Jotec E-vita Open graft (*n* = 57) or the Vascutek Thoraflex hybrid prosthesis (*n* = 11). Both, group 1 (acute aortic dissection type A and B; symptomatic penetrating aortic ulcer) and group 2 (aortic aneurysm; chronic aortic dissection) included 34 patients each.

**Results:**

Early mortality was 13.2% (14.7% in group 1 vs. 11.7% in group 2, *p* = 0.720). Neurological complications occurred in 12 patients (17.6%) (stroke: 8.8 vs. 11.7%; *p* = 0.797 and spinal cord injury: 8.8 vs. 5.9%; *p* = 0.642 in groups 1 vs. 2 respectively). Cardiopulmonary bypass time and cross clamp time were significantly longer in group 1 (252.2 ± 73.5 and 148.3 ± 34 min vs. 189.2 ± 47.8 and 116.3 ± 34.5 min; *p <  0.001*). The overall 1-, 3- and 7-year-survival was 80.9, 80.9 and 74.2% with no significant differences between groups 1 and 2. Expansion of true lumen after FET implantation was significant at all levels in both groups for patients with aortic dissection. One-, 3-, and 7-year-freedom from secondary (re-)intervention for patients for aortic dissection was 96.9, 90.2 and 82.7% with no significant differences between groups 1 and 2; *p* = 0.575.

**Conclusion:**

The FET technique can be applied in acute aortic syndromes with similar risks regarding adverse events or mortality when compared to chronic degenerative aortic disease. Postoperative increase in true lumen diameter mirrors decrease of false lumen diameter, goes along with favorable midterm outcome and prolongs freedom from secondary interventions in acute aortic dissection.

**Electronic supplementary material:**

The online version of this article (10.1186/s13019-019-0969-9) contains supplementary material, which is available to authorized users.

## Introduction

Surgical treatment of aneurysms and dissections involving the aortic arch and the descending aorta carries a significant morbidity and mortality risk. The frozen elephant trunk (FET) technique was developed to facilitate the conventional surgical two-stage approach. After its introduction as a single-stage treatment option for selected patients with aortic arch aneurysms, the indication was expanded to include patients with acute and chronic aortic dissections of DeBakey type I and also selected patients with Standford Type B aortic dissections [1–4]. Its use in the emergency setting of acute aortic dissection (AAD) is still a matter of ongoing debate [5, 6]. Meanwhile it was shown however, that the FET technique enhances stabilization of the true lumen (TL) regardless if applied in acute or chronic dissection. This aspect favors late surgical outcome [7].

The aim of this retrospective single-center study was to evaluate the operative outcome of the FET technique over a 10-year-observation period, with an additional focus on distal aortic remodeling. Patients with acute aortic disease as well as patients with chronic disease, such as atherosclerotic aneurysms involving the aortic arch and the descending aorta with or without aortic dissection were analyzed.

## Patients and methods

Between November 2006 and July 2017, 68 patients underwent aortic arch repair using the FET technique at our department. Pathologies included acute aortic dissection type A (AADA, Debakey I) and B (AADB) presenting with (multiple) intimal tears in the aortic arch and/or requiring debranching of the arch vessels to ensure adequate perfusion (group 1). Further two patients presented with acute penetrating aortic ulcers requiring acute intervention due to expanding intramural hematoma of the aortic arch segment. Patient group 2 suffered from chronic degenerative diseases, such as chronic aortic dissections of both types A and B (CADA/B), as well as thoracic aortic aneurysms.

After approval by our local ethics committee, patient data were retrieved from our prospectively maintained cardiac surgery database. All 59 patients surviving surgery and the early postoperative period (86.8% of the entire cohort), were either followed as outpatients, or were contacted to obtain complete follow-up. At least one postoperative radiological imaging study by angio-CT of the aorta was available in 88.1% of all discharged patients.

### Operative technique

All patients were operated on via median sternotomy using cardiopulmonary bypass (CPB), circulatory arrest and selective antegrade cerebral perfusion. Standard central cannulation of the ascending aorta and the right atrium was performed in the majority of patients. In 20 patients, the femoral artery or subclavian artery was cannulated. Cerebral perfusion pressure was monitored throughout the procedure by near-infrared spectroscopy (NIRS). In patients undergoing elective procedures, a cerebrospinal fluid drainage catheter was implanted before surgery for spinal cord protection by reduction of spinal pressure. Mean core cooling was accomplished by induction of moderate hypothermia at 24 °C rectal temperature. Cardiac arrest was induced after aortic cross-clamping by antegrade perfusion of the coronary arteries with cold Bretschneider’s solution (Custodiol®, Dr. Franz Köhler Chemie GmbH). Circulatory arrest was established by discontinuation of CPB. Selective antegrade cerebral perfusion was initiated after insertion of perfusion catheters in the left common carotid artery and the brachiocephalic trunk. Perfusion volume was 8–12% of the calculated cardiac output and perfusion pressure was held between 60 and 80 mmHg. Then, the FET was delivered in the descending aorta before the non-stented Dacron graft segment was sutured circumferentially to the aorta distal of the left subclavian artery. When the E-vita Open prosthesis (Jotec® Inc., Hechingen, Germany) was used, the supra-aortic vessels were implanted as a single tissue patch into a corresponding orifice incision of the vascular prosthesis. The Thoraflex™ hybrid prosthesis (Vascutek Terumo, Inchinnan, Scotland, UK) provides single side branches for each supra-aortic vessel. Proximal graft-to-aortic anastomosis at various levels of the ascending aorta completed the aortic arch repair. Only the 130 mm stented graft from Jotec (*n* = 57) and the 100 mm Thoraflex graft (*n* = 11), were used throughout the procedures.

### Radiological follow-up

All preoperative computed tomography angiograms (CTA) were compared to postoperative CTAs. The angiogram protocol routinely involved an early arterial phase and a late venous phase scan from the neck vessels to the iliac or femoral arteries.

First, the distal landing zone of the FET was identified. Furthermore, we analyzed remodeling of the true and false aortic lumen at 3 different levels in all patients with aortic dissections, using multiplanar reconstruction as previously described by Kreibich et al. [8]. The levels were defined as follows: L1 = segment at the stent level, L2 = segment between the distal end of the FET and the celiac trunk, L3 = at the level of the celiac trunk.

On each level (L1-L3) maximum total diameter of the aorta, maximum diameter of the TL, and maximum diameter of the false lumen (FL) were measured and compared to the respective preoperative values. Measurements were conducted in the plane perpendicular to the aortic center line.

We also analyzed the rate of FL thrombosis during CTA-follow-up semi-quantitatively. The degree of FL thrombosis was classified as follows: 0 = thrombosed FL, 1 = partly thrombosed FL, 2 = patent FL.

### Statistical analysis

Statistical analysis was performed using IBM SPSS Statistics version 24 software (SPSS, Chicago, IL). Normally distributed continuous variables were reported as mean ± standard deviation and were compared by two-tailed T-test. Categorical variables were reported as frequencies and percentages and were analyzed by *x* ^2^-test. Survival analysis was estimated using the Kaplan-Meier method and survival curves were compared using the Log-Rank-test. Independent risk factors were analyzed by Cox regression analysis. Variances in lumen diameter were compared by paired-T-test. The threshold for significance was set at *p* <  0.05.

## Results

Demographic data are presented in Table 1. Mean age at the time of aortic arch repair was 61.8 ± 12.9 years with no significant difference between groups. Forty-eight patients (70.6%) were male, with higher prevalence in group 1 when compared to group 2 (82.3% vs 64.5%; *p = 0.033*). In 12 patients (17.6%), a FET was implanted during cardiac re-operation. The majority of these patients had undergone previous ascending aortic repair.

**Table 1:** Patient demographics

	Total no. (%) or mean ± SD	Acute Group 1	Chronic Group 2	*p* value
Number of patients	68 (100)	34 (50)	34 (50)	
Age (y)	61.8 ± 12.9	59.0 ± 14.6	64.5 ± 10.5	0.080
Gender, male	48 (70.6)	28 (82.3)	20 (58.8)	*0.033*
Weight (kg)	80.6 ± 17.7	85.7 ± 16.2	75.6 ± 18.0	*0.018*
Height (cm)	174.5 ± 9.9	177.0 ± 8.2	171.9 ± 10.9	*0.031*
Arterial hypertension	57 (83.8)	28 (82.4)	29 (85.3)	0.742
Hyperlipidaemia	20 (29.4)	7 (20.6)	13 (38.)	0.110
Diabetes mellitus	3 (4.4)	1 (2.9)	2 (5.9)	0.555
Smoking	34 (50)	13 (38.2)	21 (61.8)	0.052
Obesity	12 (17.6)	6 (17.6)	6 (17.6)	1
COPD	5 (7.4)	2 (5.9)	3 (8.8)	0.642
Coronary artery disease	21 (30.9)	5 (14.7)	16 (47.1)	*0.004*
Renal insufficiency	5 (7.4)	2 (5.9)	3 (8.8)	0.642
Peripheral vascular disease	10 (14.7)	2 (5.9)	8 (23.5)	*0.040*
Cerebral vascular disease	8 (11.8)	2 (5.9)	6 (17.6)	0.132
Connective tissue disorder	3 (4.4)	1 (2.9)	2 (5.9)	0.555
Previous CABG	1 (1.5)	1 (2.9)	0	0.314
Previous ascending aorta replacement	8 (11.8)	0	8 (23.5)	*0.003*

*CABG* coronary artery bypass grafting, *COPD* chronic obstructive pulmonary disease

### Operative data

Operative details are shown in Table 2. All 34 patients in group 1 underwent emergency procedures. Cardiopulmonary bypass time and cross clamp time were significantly longer in patients with acute aortic syndrome, measuring 252.2 ± 73.5 and 148.3 ± 34 min in group 1 vs. 189.2 ± 47.8 and 116.3 ± 34.5 min in group 2; *p <  0.001*. Total operation time was also longer in group 1 with 402.8 ± 117.3 vs. 347.9 ± 93.0 min in group 2; *p = 0.036*. Concomitant procedures were numerically more frequent in group 1 when compared to group 2 (41.1% vs 26.4%; *p* = 0.205). In seven patients (10.3%) the aortic valve was preserved by aortic root re-implantation, while 8 (11.7%) underwent aortic valve replacement when a valve sparing approach was not possible. In another nine patients (13.2%) concomitant coronary artery bypass grafting (CABG) was necessary. Aortic valve resuspension and replacement with mechanical prothesis was performed more often when comparing groups 1 and 2 with *p*-values of *0.039* and *0.020* respectively.

**Table 2:** Peri- and postoperative data

	All patients	Acute Group 1	Chronic Group 2	*p* value
CPB (min)	220.7 ± 68.3	252.2 ± 73.5	189.2 ± 47.8	*< 0.001*
Cardioplegia (ml)	1750.7 ± 620.0	1994.1 ± 417.1	1507.4 ± 696.1	*0.001*
Cross clamp time (min)	132.3 ± 38.8	148.3 ± 34.5	116.3 ± 34.5	*< 0.001*
Circulatory arrest (min)	58.8 ± 35.1	62.4 ± 37.4	54.9 ± 37.4	0.385
Reperfusion (min)	65.1 ± 33.5	75.4 ± 40.0	55.1 ± 21.6	*0.012*
Hypothermia (°C)	23.8 ± 3.2	23.3 ± 3.8	24.3 ± 2.4	0.189
Operation time (min)	375.4 ± 108.6	402.8 ± 117.3	347.9 ± 93.0	*0.036*
Blood transfusion (ml)	2116.9 ± 2045.3	2550.0 ± 2471.7	1683.8 ± 1413.3	0.082
Plasma transfusion (ml)	1288.5 ± 1492.2	1514.7 ± 1701.8	1062.4 ± 1232.5	0.214
Jotec prosthesis^a^	57 (83.8)	31 (91.2)	26 (76.5)	0.100
Thoraflex prosthesis^b^	11 (16.2)	3 (8.8)	8 (23.5)	0.100
Prosthesis diameter	26.0 ± 3.2	26.1 ± 2.4	25.9 ± 3.9	0.766
Central aortic cannulation	48 (70.6)	18 (52.9)	30 (88.2)	*0.001*
Right femoral cannulation	10 (14.7)	8 (23.5)	2 (5.9)	*0.040*
Left femoral cannulation	3 (4.4)	2 (5.9)	1 (2.9)	0.555
Right subclavian cannulation	7 (10.3)	6 (17.6)	1 (2.9)	*0.046*
Ascending aorta replacement	54 (79.4)	31 (91.2)	23 (67.6)	*0.016*
Valve reconstruction after David	3 (4.4)	2 (5.9)	1 (2.9)	0.555
Aortic valve resuspension	4 (5.9)	4 (11.8)	0	*0.039*
Mechanical AVR	5 (7.4)	5 (14.7)	0	*0.020*
Biological AVR	3 (4.4)	2 (5.9)	1 (2.9)	0.555
CABG	9 (13.2)	2 (5.9)	7 (20.6)	0.074
ICU (d)	9.0 ± 9.3	8.7 ± 9.3	9.2 ± 9.4	0.826
IMC (d)	3.7 ± 4.4	3.1 ± 4.0	4.4 ± 4.7	0.246
Intubation (h)	121.4 ± 166.0	94.9 ± 117.9	147.8 ± 201.6	0.191
Hospital stay (d)	24.2 ± 26.0	18.8 ± 12.9	29.5 ± 33.8	0.092
Bleeding	17 (25)	8 (23.5)	9 (26.5)	0.779
Re-Thoracotomy for bleeding	4 (5.9)	1 (2.9)	3 (8.8)	0.303
Spinal Cord injury	5 (7.4)	3 (8.8)	2 (5.9)	0.642
Cerebrovascular injury	7 (10.3)	3 (8.8)	4 (11.7)	0.797
Recurrens paresis	9 (13.2)	2 (5.9)	7 (20.6)	0.074
Phrenicus paresis	3 (4.4)	0	3 (8.8)	0.076
ACRF	39 (57.4)	23 (67.6)	16 (47.1)	0.086
Dialysis	18 (26.5)	10 (29.4)	8 (23.5)	0.582
Re-intubation	11 (16.2)	3 (8.8)	8 (23.5)	0.100

*ACRF* acute on chronic renal failure, *AVR* aortic valve replacement, *CABG* coronary artery bypass grafting, *CPB* cardiopulmonary bypass, *ICU* intensive care unit, *IMC* intermediate care unit

^a^ Jotec E-vita Open, ^b^ Vascutek Thoraflex hybrid

The mean diameter of the implanted stent segment of the hybrid graft was 26.0 ± 3.2 mm with no differences between groups 1 and 2.

### Postoperative outcome and clinical follow-up

**Fig. 1:**
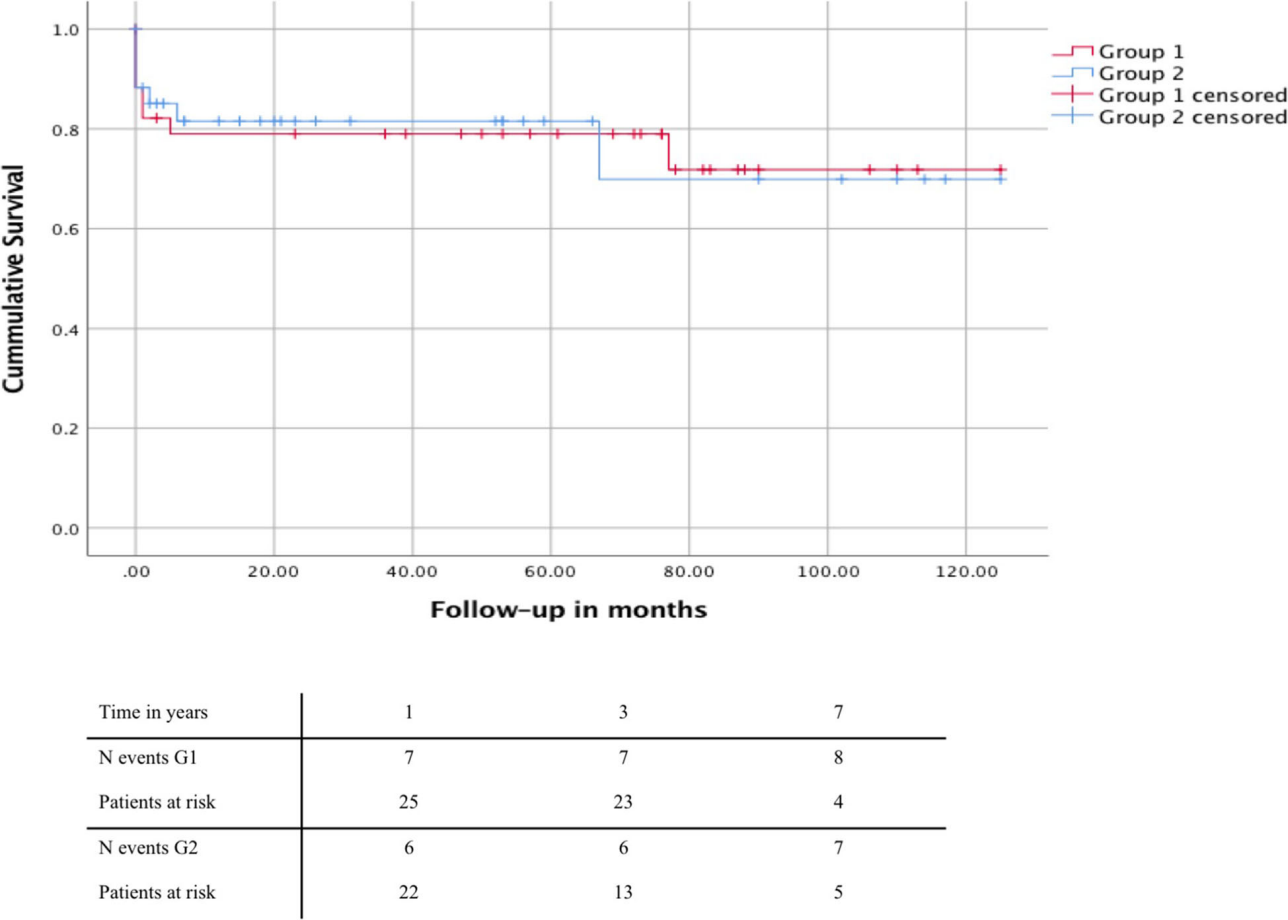
Kaplan-Meier Analysis of overall survival for patients after FET. Depiction of survival in patients of group 1 vs. group 2

Overall early mortality was 13.2% (9 patients); with 14.7% (5 patients) in group 1 and 11.7% (4 patients) in group 2; *p* = 0.720. The overall 1-, 3- and 7-year-survival was 80.9, 80.9 and 74.2% with no significant difference between groups 1 and 2 (Fig. 1).

Neurological events (stroke or transient ischemic attack) occurred in 7 patients (10.3%), and five patients (7.4%) suffered from postoperative spinal cord injury (SCI), with permanent paraplegia in 3 patients (4.4%). Stroke rate was comparable in both groups with 3 patients (8.8%) in group 1 and 4 patients (11.7%) in group 2; *p* = 0.797. Three patients (8.8%) in group 1 and 2 patients (5.9%) in group 2 developed SCI *p* = 0.642. Peri- and postoperative parameters are summarized in Table 2. Detailed outcome according to pathology are presented in Additional file 1: Table S1.

Second-stage procedures in the descending and abdominal aorta were performed in 18 patients (26.5%). In 15 cases (83.3%) transfemoral endovascular interventions became necessary, while 3 patients (16.6%) had to undergo conventional open surgery on the downstream aorta. Three patients (20.0%) in the endovascular intervention group needed additional open abdominal aortic (bypass) surgery subsequently.

Subgroup analysis revealed 6 patients (17.6%) in group 1, who required further procedures on the downstream aorta. Of those however, only two patients (8.0%) with previous AADA needed secondary surgery during follow-up. One patient was treated with an endovascular stent graft implantation, including proximal extension of the stented segment of the FET by overstenting of the left subclavian artery. The other patient received an open repair with the Gelweave™-Coselli-prosthesis (Vascutek Terumo, Inchinnan, Scotland, UK), due to extensive aortic pathology (Loeys-Dietz Syndrome).

Another 12 patients (35.3%) in group 2 underwent secondary interventions. Nine patients (26.5%) received secondary completion for thoraco-abdominal aneurysm repair, while three patients (8.8%) underwent re-interventions for remaining chronic aortic dissection type A and B. One patient received an open bypass graft to the celiac trunk, 1 patient received kissing stent implantation in the iliac arteries and seven patients were treated by thoracic endovascular stent graft implantation with extension of the FET into the descending aorta. Three patients needed a combined completion with downstream aortic replacement by endovascular stent graft implantation and bypass surgery to visceral arteries.

**Fig. 2:**
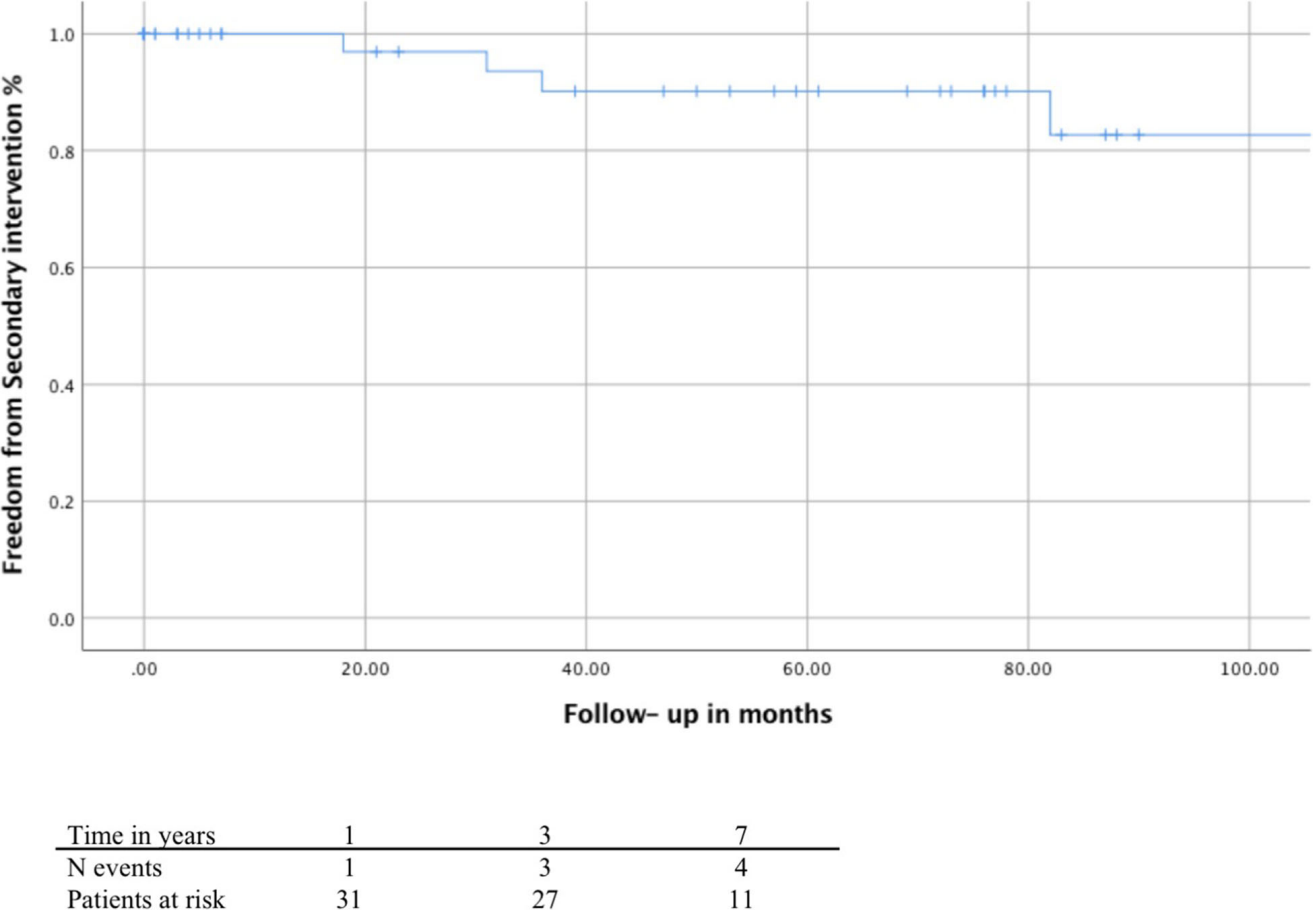
overall freedom from (re-)intervention for patients with previous aortic dissection. Overall freedom from re-intervention for patients who were discharged

One-, 3-, and 7-year-freedom from secondary (re-)intervention for patients with aortic dissection was 96.9, 90.2 and 82.7%, showing no significant differences between groups 1 and 2; *p* = 0.575 (Fig. 2).

The variables reperfusion time, amount of blood transfusions during surgery, duration of intermediate care stay, intubation time, incidence of re-thoracotomy and perioperative dialysis were correlated with overall mortality in univariate regression analysis. Upon subsequent multivariate regression analysis, only reperfusion time was found to be an independent predictor of mortality, with a hazard ratio (HR) of 1.026 (95% CI1.002–1.049; *p = 0.031*).

### Radiological follow-up

Postoperative CTAs were assessed in 52 out of the 59 discharged patients (88.1%). Mean follow-up time between CTA scans in group 1 was 653.2 ± 760 days vs. 305.2 ± 398 days in group 2; *p* = 0.196. The overall mean level of the distal end of the stented graft was at thoracic vertebra (T) 8.5 ± 1.4 with no significant differences between groups 1 and 2. Regression analysis showed no correlation between the level of distal landing zone and the occurrence of SCI.

For the effect on aortic remodeling after aortic dissections types A and B, CTA analysis included 26 patients who were operated on in group 1 (*n* = 16) and group 2 (*n* = 10).

The differences in total aortic diameter, TL and FL diameter, as well as FL thrombosis were analyzed at the aforementioned levels.

#### Segment at the stent level L1

**Fig. 3:**
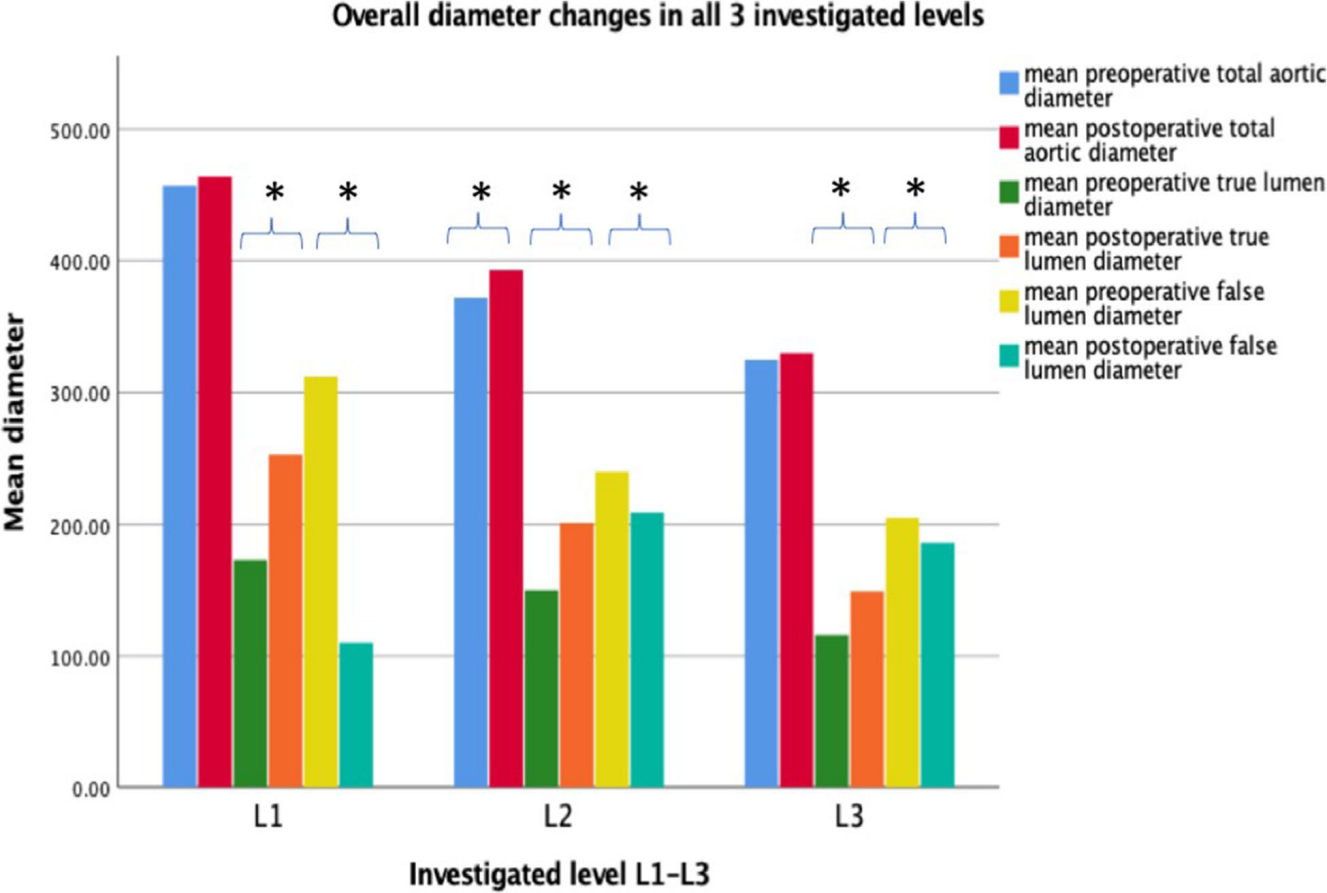
overall changes in aortic diameters for patients with previous aortic dissection. Illustration of pre- and postoperative diameter changes for patients with previous aortic dissection of mean aortic diameter, true lumen (TL) and false lumen (FL) at the different levels L1-L3 for patients discharged. Significance in diameter changes are marked with asterisk

The mean total aortic diameter did not vary significantly between pre- and postoperative CTAs in all patients (45.8 ± 14.0 mm vs. 45.7 ± 15.7 mm; *p* = 0.962). However, all patients, whether with acute or chronic dissection, presented a significant increase in TL diameter (16.9 ± 5.1 mm vs. 25.2 ± 3.3 mm; *p <  0.001*) and decrease of FL diameter (32.4 ± 12.7 mm vs. 10.6 ± 12.4 mm; *p < 0.001*) after the operation (Fig. 3).

Interestingly, there was a significant decrease in total diameter in patients of group 2, compared to a slight increase in group 1 after the procedure: − 4.1 ± 4.9 mm vs. 2.7 ± 3.7 mm; *p = 0.002*.

**Fig. 4:**
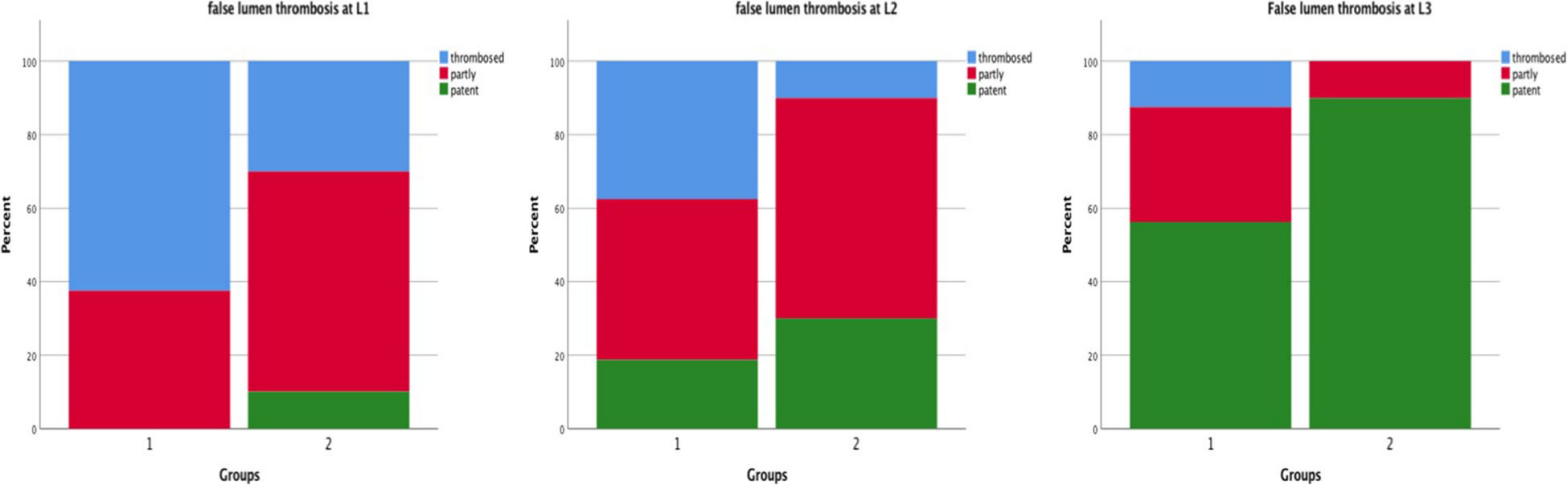
patency of false lumen for patients with previous dissection. Difference in aortic false lumen thrombosis in radiological follow-up CTAs for patients who were treated for acute or chronic aortic dissection after hospital discharge

Complete thrombosis of the FL was found more often in group 1 (10 patients, 62.5%) when compared to group 2 (3 patients, 30*%), p* = 0.090. While false lumen patency was found in 1 (10%) patient in group 2, patients in group 1 had either complete or partial FL thrombosis at L1 (Fig. 4).

#### Segment from the distal end of the FET down to the celiac trunk L2

The mean total aortic diameter increased significantly at L2 in all assessed patients from 36.7 ± 10.8 mm to 38.5 ± 11.4; *p = 0.012*. Again, there was a significant increase in TL diameter (14.6 ± 6.0 mm vs. 20.0 ± 6.1.mm; *p < 0.001*) and decrease of FL diameter (24.9 ± 11.4 mm vs. 20.8 ± 12.3 mm; *p = 0.036*) (Fig. 3). The changes in lumen diameters between groups 1 and 2 did not reach statistical significance.

In group 1, complete thrombosis of FL was observed in six patients (37.5%), seven patients (43.8%) had their FL partially thrombosed and another three patients (18.8%) showed a patent FL. In group 2, 1 patient (10%) presented with completely thrombosed FL at L2, 6 patients (60%) had a partly thrombosed FL, and 3 patients (30%) presented a patent FL during CTA-follow-up; *p* = 0.172 (Fig. 4).

#### Segment at the celiac trunk L3

Similar to L1, the mean total aortic diameter remained constant at L3 in all analyzed CTAs (32.1 ± 4.9 mm vs. 32.6 ± 4.9 mm; *p* = 0.175). The increase in TL diameter (11.2 ± 4.5 mm vs. 14.7 ± 3.5 mm; *p < 0.001*) and decrease of FL diameter (21.3 ± 7.1 mm vs. 18.4 ± 7.5 mm; *p = 0.005*) was statistically significant (Fig. 3).

There were no significant changes in diameter between patients within both groups.

In group 1, complete thrombosis of FL was seen in two patients (12.5%), five patients (31.3%) had a partly thrombosed FL and another nine patients (56.3%) remained with a patent FL. On the other hand, no patient in group 2 presented with complete thrombosis of FL at L3 during follow-up. One patient (10%) showed a partly thrombosed FL, and in the remaining 9 patients (90%) a patent FL was present; *p = 0.036* (Fig. 4).

## Discussion

We investigated early- and midterm results of the FET technique over a 10-year-obeservation period at our clinic. This modification of the classical elephant trunk technique has emerged as a valuable treatment option simplifying the repair of aneurysms involving the aortic arch and the proximal descending aorta, significantly [4, 9]. It provides the means for a single-stage treatment and enables further downstream aortic repair, through secondary interventions by conventional or endovascular surgery [5, 10–12]. After its original implementation in patients with aortic aneurysm and CADA/B, it was also applied in patients with complicated AADA/B. Even though there is an ongoing discussion, whether this radical and extensive repair should be recommended in the acute setting generally, it promotes instant TL perfusion of the descending aorta, thereby reducing the risks associated with TL collapse. In addition, mechanical stabilization of the TL counteracts FL perfusion and expansion [5].

In this study we performed an outcome analysis of our patients, either presenting with acute or chronic aortic dissection and non-dissecting aortic aneurysm. Knowing that these patients present with different underlying disease, we aimed to investigate variables, possibly affecting distal aortic remodeling.

Our analysis showed comparable overall outcome results in both groups regarding survival and adverse clinical events. Early mortality for all patients was 13.2% which is in line with previous reports ranging between 7.8 and 17.2% mortality rates [4, 13–15]. There was no significant difference in the early mortality rate between the acute and chronic group (14.7 vs 11.7%, *p* = 0.720). With regard to the underlying aortic disease, the mortality rates were also comparable to those reported in the literature [14]. Multivariate analysis revealed no significant independent risk factors for early mortality. Nonetheless, in multivariate regression analysis the variable “reperfusion time” was identified as an independent risk factor for overall mortality with an HR of 1.026. In our study cohort, prolonged reperfusion time was necessary in complex surgical procedures, requiring additional cardiac interventions. This variable therefore appears as a surrogate parameter, reflecting an increased risk associated with more demanding, concomitant procedures.

The incidence of neurological complications is of particular interest, since this adverse event is likely to increase morbidity and mortality [16]. Contemporary results for conventional aortic arch surgery indicate stroke rates ranging between 5 and 14% [14, 17–19]. In patients undergoing the FET technique in the acute setting, they have been reported to be even higher [5]. In our series, 10.3% of patients developed postoperative stroke with disabling neurological deficits. There were no significant differences between the acute and chronic patient group. Different cannulation sites in the acute setting did not correlate with the occurrence of postoperative stroke in our patient cohort. Recently, central ascending aortic, axillary, and femoral cannulation in AADA patients were compared, with no differences in the occurrence of postoperative stroke detected [20].

Another serious neurological sequel is the development of SCI. Especially in the acute setting, the effect and duration of malperfusion before aortic repair and restoration of antegrade perfusion is poorly understood. The respective incidences are reported to range between 4 and 9% in accordance to the underlying aortic disease [17, 19, 21–23]. In the present study SCI occurred in 5 patients (7.4%) with no significant difference regarding the underlying aortic pathology, similar to previous reports by Leontyev et al. [14]. In contrast to their study however, we found no correlation between core body temperature, circulatory arrest time or the distal landing zone level of the FET and the occurrence of SCI. This finding might possibly be due to the use of shorter prosthesis lengths in our patient groups, as reflected by the more upstream ending of the stented segment between T8 and T9.

The majority of procedures were performed -as expected- in male patients (70.6%), since the incidence of thoracic aortic disease is known to be lower in the female gender [24]. Interestingly, the male to female ratio was significantly higher in group 1 when compared to group 2. Whether gender differences play a role in treatment outcome of AADA as reported in aortic aneurysms needs further investigation [25].

In DeBakey Type I AADA, replacement of the ascending aorta and the proximal arch segment only, may leave remaining entry sites in the downstream aorta untreated. Risks associated with persisting re-entries within the aortic arch and beyond are thereby increased. In up to 90% of these patients, long-term FL patency is reported after this conservative, less radical surgical approach [21]. In addition, when remaining re-entries in the aortic arch promote expansion of the FL, further re-interventions may become necessary [15, 26–28]. By applying the more demanding FET technique in this particular setting instead, the aortic arch and the proximal descending aorta are both secured. A distal landing zone is provided for future interventions. Later postoperative distal aortic dilatation is more likely to be prevented in the majority of these patients. Previous reports also indicated that this effect is accompanied by the expansion of TL further downstream, on the expense of the respective FL diameter [15, 29]. Our follow-up CTA analysis verified an augmentation in the TL diameter, while corresponding FL diameter decreased at all 3 reviewed levels. With no changes in the total diameter of the aorta. The positive effect on diameter changes did not differ significantly between acute and chronic aortic dissections. In addition, freedom from aortic re-interventions was satisfactory with over 80% at 7 years, for all patients. Furthermore, when secondary re-interventions became necessary, most patients (83.3%) were successfully treated through an endovascular approach. Moreover, the difference between groups 1 and 2 with respect to re-interventions, was not significant. This observation supports the potential benefit of the more aggressive surgical approach in the acute setting, when only addressing the ascending aorta and proximal aortic arch segment seem insufficient to secure antegrade TL perfusion. Enhancement of TL perfusion, supported in turn the positive remodeling of the descending aorta.

Immer et al. [30] found that the area most likely to develop late aneurysm is the proximal descending aorta, due to higher shear stress in the convexity of the aorta. Using the FET technique, potential intimal tears in the proximal descending aorta are sealed by the stented segment of the FET. Compression of the FL facilitates expansion of the TL. Driving forces for further dilatation of the proximal descending aorta are hence reduced [10, 30]. Due to the recency of the process in the acute setting, the observed number of patients with complete or partial FL thrombosis was higher in patients with AADA/B when compared to patients with CADA/B in our study. In patients with chronic dissection, progressive intimal thickening and the presence of multiple re-entry tears distal of the stent-graft may prevent complete occlusion of the FL [29]. This is reflected by the significantly increased incidence of FL thrombosis in group 1 compared to group 2 at L3.

Our data suggest that early FL thrombosis is enabled by FET-induced TL expansion and perfusion enhancement. Therefore, the risks associated with further expansion and subsequent need for secondary downstream aortic interventions become less likely. This fact further delineates the advantages of the more extensive repair using the FET technique in the acute setting for patients with evident malperfusion and extensive involvement of the arch segment. Nevertheless, it needs to be noted that the accompanied risk for adverse neurological events associated with this more demanding repair, need to be carefully weighed against the potential benefits of decreased aortic dilation and re-interventions. Although the majority of FET procedures are currently performed in patients with DeBakey Type I AAD, a significant proportion of these patients still undergo hemi- or partial-arch repair. The indication whether to extend this form of lifesaving surgery to the more complex FET, must be judiciously applied to patients presenting risks for developing late events.

## Conclusion

The FET procedure provides satisfactory early and mid-term results for the treatment of extensive aortic disease. The technique offers a safe alternative in the acute setting, with no significant differences with regard to adverse events or mortality compared to elective cases. Extensive repair by FET in the acute and chronic setting has the potential to reduce late aortic events through favorable aortic remodeling along the downstream aorta. The increase in TL diameter and decrease of FL perfusion provides positive long-term outcomes and prolongs the freedom from secondary interventions in acute aortic dissections.

### Limitations

Main limitations of this study are its retrospective nature, small sample size and being performed in a single-center.

## Additional file

Additional file 1: Detailed outcome according to underlying aortic pathology. (DOCX 25 kb)

## Data Availability

Supporting data are available upon request through the corresponding author. Publication of raw data is not possible as it will conflict with our privacy policy.

## Abbreviations

AAD: Acute aortic dissection
AADA/B: Acute aortic dissection type A or B
CABG: coronary artery bypass grafting
CADA/B: chronic aortic dissection type A or B
CPB: Cardiopulmonary bypass
CTA: Computed tomography angiograms
FET: Frozen elephant trunk
FL: False lumen
NIRS: Near-infrared spectroscopy
SCI: Spinal cord injury
TL: True lumen
