# Supplementary material for: Aortic arch replacement with frozen elephant trunk technique – a single-center study
Source: J Cardiothorac Surg. 2019 Aug 1;14:147. doi: 10.1186/s13019-019-0969-9 (PMC6676558; doi:10.1186/s13019-019-0969-9)
Supplement: Supplementary file 1 — Detailed outcome according to underlying aortic pathology. (DOCX 25 kb) [file 13019_2019_969_MOESM1_ESM.docx]

Additional file 1

Table S1

|  | AADA  n=25 | AADB  n=6 | PAU  n=2 | CADA  n=8 | CADB  n=6 | TAAA  n=19 | other  n=2 |
| --- | --- | --- | --- | --- | --- | --- | --- |
| ICU (d) | 9.3 ± 10.4 | 8.3 ± 6.2 | 3.0 ± 0.0 | 11.8 ± 11.5 | 9.5 ± 13.3 | 8.5 ±7.3 | 4.0 ± 4.2 |
| IMC (d) | 3.8 ± 4.4 | 1.3 ± 1.2 | 1.0 ± 1.4 | 5.1 ± 6.2 | 3.8 ± 4.4 | 4.4 ± 4.3 | 1.0 ± 1.4 |
| Intubation (h) | 105.8 ± 133.2 | 81.0 ± 56.2 | 27.5 ± 21.9 | 178.3 ± 225.8 | 207.3 ± 298.1 | 120.1 ± 165.1 | 57.0 ± 22.6 |
| Hospital stay (d) | 19.3 ± 13.1 | 20.7 ± 14.7 | 10.0 ± 9.8 | 44.4 ± 62.5 | 22.2 ± 11.9 | 26.9 ± 19.5 | 8.5 ± 6.4 |
| Bleeding | 6 (24.0) | 2 (33.3) | 0 | 3 (37.5) | 0 | 5 (26.3) | 1 (50.0) |
| Re-Thoracotomy for bleeding | 1 (4.0) | 0 | 0 | 0 | 0 | 2 (10.5) | 1 (50.0) |
| Spinal Cord injury | 2 (8.0) | 1 (16.6) | 0 | 2 (25) | 0 | 0 | 0 |
| Cerebrovascular injury | 4 (16.0) | 1 (16.6) | 1 (50.0) | 1 (12.5) | 1 (16.6) | 4 (21.1) | 0 |
| Recurrens paresis | 2 (8.0) | 0 | 0 | 3 (37.5) | 0 | 4 (21.1) | 0 |
| Phrenicus paresis | 0 | 0 | 0 | 2 (25.0) | 0 | 1 (5.3) | 0 |
| ACRF | 18 (72.0) | 5 (83.3) | 0 | 2 (25.0) | 1 (16.6) | 12 (63.2) | 1 (50.0) |
| Dialysis | 8 (32.0) | 2 (33.3) | 0 | 2 (25.0) | 1 (16.6) | 4 (21.1) | 1 (50.0) |
| Re-intubation | 3 (12.0) | 0 | 0 | 1 (12.5) | 1 (16.6) | 5 (26.3) | 1 (50.0) |
| AADA= acute aortic dissection type A, AADB= acute aortic dissection type B, PAU= penetrating aortic ulcer, CADA= chronic aortic dissection type A, CADB= chronic aortic dissection type B, TAA= thoracic aortic aneurysm, other= 1 aortic plaque rupture + 1 aortic valve endocarditis re-do operation  ICU= intensive care unit, IMC= intermediate care unit, ACRF= acute on chronic renal failure | | | | | | | |

Table S2

|  | Acute aortic dissections A+B  n=31 | Chronic aortic dissections A+B  n=14 | p-value |
| --- | --- | --- | --- |
| ICU (d) | 9.1 ± 9.7 | 10.8 ± 11.8 | 0.651 |
| IMC (d) | 3.3 ± 4.1 | 4.6 ± 5.4 | 0.437 |
| Intubation (h) | 101.0 ± 121.8 | 190.7 ± 248.7 | 0.218 |
| Hospital stay (d) | 19.6 ± 13.2 | 34.9 ± 47.8 | 0.260 |
| Bleeding | 8 (25.8) | 3 (21.4) | 0.752 |
| Re-Thoracotomy for bleeding | 1 (3.2) | 0 | 0.497 |
| Spinal Cord injury | 3 (9.7) | 2 (14.3) | 0.649 |
| Cerebrovascular injury | 5 (16.1) | 2 (14.3) | 0.874 |
| Recurrens paresis | 2 (6.5) | 3 (21.4) | 0.139 |
| Phrenicus paresis | 0 | 2 (14.3) | *0.031* |
| ACRF | 23 (74.2) | 3 (21.4) | *0.001* |
| Dialysis | 10 (32.3) | 3 (21.4) | 0.458 |
| Re-intubation | 3 (9.7) | 2 (14.3) | 0.649 |
| ICU= intensive care unit, IMC= intermediate care unit, ACRF= acute on chronic renal failure | | | |
